# Repetitive Transcranial Magnetic Stimulation With and Without Internet-Delivered Cognitive-Behavioral Therapy for the Treatment of Resistant Depression: Protocol for Patient-Centered Randomized Controlled Pilot Trial

**DOI:** 10.2196/18843

**Published:** 2020-10-27

**Authors:** Rabab M Abou El-Magd, Gloria Obuobi-Donkor, Medard K Adu, Christopher Lachowski, Surekha Duddumpudi, Mobolaji A Lawal, Adegboyega O Sapara, Michael Achor, Maryam Kouzehgaran, Roshan Hegde, Corina Chew, Mike Mach, Shelley Daubert, Liana Urichuk, Mark Snaterse, Shireen Surood, Daniel Li, Andrew Greenshaw, Vincent Israel Opoku Agyapong

**Affiliations:** 1 Department of Psychiatry Faculty of Medicine and Dentistry University of Alberta Edmonton, AB Canada; 2 Addiction and Mental Health Alberta Health Services Edmonton, AB Canada

**Keywords:** repetitive transcranial magnetic stimulation, internet-delivered cognitive-behavioral therapy, treatment of resistant depression, cognitive-behavioral therapy, depression

## Abstract

**Background:**

Major depression is a severe, disabling, and potentially lethal clinical disorder. Only about half of patients respond to an initial course of antidepressant pharmacotherapy. At least 15% of all patients with major depressive disorder (MDD) remain refractory to any treatment intervention. By the time that a patient has experienced 3 definitive treatment failures, the likelihood of achieving remission with the fourth treatment option offered is below 10%. Repetitive transcranial magnetic stimulation (rTMS) is considered a treatment option for patients with MDD who are refractory to antidepressant treatment. It is not currently known if the addition of internet-delivered cognitive-behavioral therapy (iCBT) enhances patients’ responses to rTMS treatments.

**Objective:**

This study will evaluate the initial comparative clinical effectiveness of rTMS with and without iCBT as an innovative patient-centered intervention for the treatment of participants diagnosed with treatment-resistant depression (TRD).

**Methods:**

This study is a prospective, two-arm randomized controlled trial. In total, 100 participants diagnosed with resistant depression at a psychiatric care clinic in Edmonton, Alberta, Canada, will be randomized to one of two conditions: (1) enrolment in rTMS sessions alone and (2) enrolment in the rTMS sessions plus iCBT. Participants in each group will complete evaluation measures (eg, recovery, general symptomatology, and functional outcomes) at baseline, 1 month, 3 months, and 6 months. The primary outcome measure will be the mean change to scores on the Hamilton Depression Rating Scale. Patient service utilization data and clinician-rated measures will also be used to gauge patient progress. Patient data will be analyzed with descriptive statistics, repeated measures, and correlational analyses.

**Results:**

We expect the results of the study to be available in 24 months. We hypothesize that participants enrolled in the study who receive rTMS plus iCBT will achieve superior outcomes in comparison to participants who receive rTMS alone.

**Conclusions:**

The concomitant application of psychotherapy with rTMS has not been investigated previously. We hope that this project will provide us with a concrete base of data to evaluate the practical application and efficacy of using a novel combination of these two treatment modalities (rTMS plus iCBT).

**Trial Registration:**

ClinicalTrials.gov NCT0423965; https://clinicaltrials.gov/ct2/show/NCT04239651

**International Registered Report Identifier (IRRID):**

PRR1-10.2196/18843

## Introduction

### Background and Rationale

Major depression is a severe, disabling, and potentially lethal clinical disorder [1-3]. Although there are a wide variety of pharmaceutical agents available as treatments for major depression, only about half of patients respond to an initial course of antidepressant pharmacotherapy [4,5]. For these patients, the current standard of care involves an empirical series of treatment attempts, typically using medication switches, antidepressant combinations, or adjunctive therapy with mood stabilizers, benzodiazepines, atypical antipsychotics, or other agents [6]. The adverse event burden and tolerability of some of these more complex interventions are not trivial and are a significant factor that hinders patient adherence to treatment [7]. Similarly, although there is increasing evidence that at least some of the atypical antipsychotics are effective as adjuncts to antidepressants, the potential for side effects, including weight gain and dyslipidemia, warrants both caution and careful clinical management.

It has been conservatively estimated that at least 15% of all patients with major depressive disorder (MDD) remain refractory to any treatment intervention [5,6]. Although a complicated relationship exists between disease chronicity and ineffective treatment [8], clinical evidence suggests that the higher the number of treatment failures, the lower the likelihood of good treatment response to subsequent interventions [4,9]. The reported results of the STAR*D study are the most vivid example of this clinical phenomenon [10-16]. In that work, there was an increased likelihood of more reduced response with each successive treatment failure. For example, after the first treatment attempt, about 30% of patients remitted. By the time that a patient had experienced 3 definitive treatment failures, the likelihood of achieving remission with the fourth treatment option offered fell below 10%. Poor treatment adherence and high discontinuation rates represent a major challenge, particularly for pharmacotherapy. Strategies for enhancing adherence include patient education and supported self-management, as well as the use of collaborative care systems by practitioners. Treatment adherence should be discussed at an early stage and monitored frequently during treatment in a collaborative manner. A weak therapeutic alliance predicts poorer treatment adherence [17]. These facts underline the clinical urgency for physicians to identify treatment-resistant patients as early as possible so that alternative treatments with proven efficacies may be offered sooner. In turn, this will result in superior treatment outcomes for these treatment-resistant patients.

Technology and the internet have dramatically changed medicine. According to Statistics Canada, 83% of Canadians had internet access in 2012, and more than 70% use the internet daily; in addition, 62% were smartphone users [17]. E–mental health refers to the use of computers, internet, and mobile devices for mental health information and care provision [18]. E–mental health apps are now widely available for information, screening, assessment and monitoring, interactive self-management, psychotherapy, and social support. Clinicians should be aware that there are benefits and potential harms to using and recommending e–mental health apps and that few have good-quality evidence of effectiveness [18-20]. Meta-analyses and reviews of computer-based psychological treatment for the treatment of MDD, whether delivered over the internet or as a stand-alone program, demonstrate convincing support for these treatment modalities [21-27]. Internet- and computer-delivered cognitive behavioral therapy (iCBT) can also be helpful in relapse prevention [28].

In 2009, the Canadian Network for Mood and Anxiety Treatments (CANMAT), a not-for-profit scientific and educational organization, published a revision of evidence-based clinical guidelines for the treatment of depressive disorders [29]. CANMAT updated these guidelines in 2016 to reflect new evidence in the field [30-35]. These updated CANMAT guidelines cover a variety of treatments, including psychological treatments in general and cognitive-behavioral therapy (CBT) in particular, as well as pharmacological treatments, neurostimulation, and complementary and alternative medicine (CAM) treatments. Choosing a first-line treatment among these treatment choices remains a collaborative decision between patient and clinician. However, there continues to be greater evidence and clinical experience with traditional treatments (psychotherapy and pharmacotherapy) and few studies directly comparing these with neurostimulation or CAM treatments. In addition, many studies of neurostimulation include populations of patients who have failed at least one previous treatment. Therefore, first-line psychological and/or pharmacological treatments should usually be considered before neurostimulation or CAM treatments [17,31-35].

Neurostimulation, also referred to as neuromodulation, is an expanding area of research and clinical interest, driven in part by the increasing knowledge base on the neurocircuitry of depression [36]. Most of these neurostimulation treatments have been studied and are used in patients with TRD who have failed to respond to standard treatments [33]. However, no previous studies examined the effect of rTMS plus iCBT in comparison to rTMS alone. Our study hypothesis is to enhance the efficiency of the treatment and assess the initial comparative clinical effectiveness of rTMS treatments when used with and without iCBT in a patient population where an improvement in treatment effects is much needed.

### Repetitive Transcranial Magnetic Stimulation

rTMS uses powerful (1.0-2.5 Tesla) focused magnetic field pulses to induce electrical currents in neural tissue noninvasively via an inductor coil placed against the scalp. Therapeutic rTMS is usually delivered by a trained technician or nurse under physician supervision. Unlike electroconvulsive therapy (ECT), no anesthesia is required. The therapeutic mechanism of rTMS is still under investigation, with mechanisms proposed at molecular, cellular, and network levels [37]. Standard protocols deliver rTMS once daily, 5 days/week. Stimulation 3 times/week has been reported as similarly effective, albeit with slower improvement and a similar number of sessions required overall [38]. “Accelerated” protocols with multiple daily sessions (2-10/day) are being explored to complete the course more rapidly [39,40]. Repeated rTMS sessions can exert therapeutic effects lasting several months [33]. Clinical trials and naturalistic studies have found maximal effects at 26-28 sessions [41,42]. Clinical experience concurs in suggesting 20 sessions before declaring treatment failure, with extension to 25-30 sessions if improvements occur [33].

More than 30 systematic reviews and meta-analyses have been conducted on rTMS in depression, with most studies involving participants with some degree of treatment resistance (ie, having failed at least 1 or 2 antidepressant trials). Overall, rTMS is considered a first-line treatment for MDD for participants who have failed at least 1 antidepressant treatment. Both high-frequency (10 Hz) rTMS of the left dorsolateral prefrontal cortex (DLPFC) and low-frequency (1 Hz) rTMS of the right DLPFC have demonstrated efficacy in numerous meta-analyses [43-46], with no differences in outcomes between them [43]. Hence both high-frequency left DLPFC and low-frequency right DLPFC are first-line rTMS protocol recommendations.

The efficacy of rTMS is established in patients with TRD defined by stringent criteria [47]. The most recent meta-analysis of high-frequency left DLPFC rTMS for TRD (23 trials, n=1156) illustrated significant efficacy of rTMS over sham, with a weighted mean difference of 2.31 and an effect size of 0.33 [48]. In addition, randomized controlled trials (RCTs) with adequate sessions (20-30) and treatment durations of 4 weeks or more achieved 40%-55% response and 25%-35% remission rates, and a real-world effectiveness study reported 58% response and 37% remission rates [42]. Similarly, for low-frequency right DLPFC rTMS, a meta-analysis (8 trials, n=263) revealed that patients who received the treatment had superior remission rates compared to sham (35% versus 10%, respectively, *P*<.001) [49]. Maintenance treatment is essential to prevent relapse following successful rTMS sessions. One study (n=204) reported median relapse time at 120 days, with relapse rates of 25%, 40%, 57%, and 77% at 2, 3, 4, and 6 months, respectively [50]. In another study (n=257), maintenance rTMS sessions were needed over 12 months for sustained remission in 71% of rTMS remitters and response in 63% of rTMS responders [51]. Moreover, a study found that without maintenance, 38% of rTMS responders relapsed within 24 weeks, at a mean of 109 days posttreatment [52]. With reintroduction of rTMS as needed, 73% met response criteria and 60% met remission criteria at 24 weeks [52]. Various rTMS maintenance schedules have been proposed [53,54], yet there is insufficient evidence to support any particular schedule of maintenance sessions.

### Cognitive-Behavioral Therapy

Cognitive-behavioral therapy is an evidence-based, structured, intensive, time-limited, symptom-focused form of psychotherapy recommended for the treatment of major depression and anxiety disorders [55]. Internet-delivered CBT (iCBT) is structured CBT delivered via the internet. CBT helps people become aware of how certain negative automatic thoughts, attitudes, expectations, and beliefs contribute to feelings of sadness and anxiety. Specifically, “people undergoing CBT learn how their thinking patterns, which may have developed in the past to deal with difficult or painful experiences and negatively affect their behavior, can be identified and changed to reduce unhappiness” [56].

Barriers to conventional face-to-face treatment include stigmas around people seeking help in person, geography (distance from a health care professional), time, and cost. Increasingly, there is a desire to pursue internet delivery as an option to increase access to treatment [57].

ICBT consists of structured modules with clearly defined goals and is delivered via the internet [56]. Although there are many types of iCBT programs, each is a goal-oriented session that typically consists of 8-12 modules and can be guided or unguided [56]. ICBT programs are made available by computer, smartphone, or tablet, for a fee [56]. With unguided iCBT, participants are informed of a website through which they can participate in an online self-directed program. Guided iCBT involves support from a regulated health professional (eg, social worker, psychologist, psychotherapist, occupational therapist, nurse, or physician). In guided iCBT, people complete modules and communicate (via email, text messages, or telephone calls) their progress to a regulated health care professional [56].

MoodGYM is the iCBT program that will be used in this study. Its stated aims are to help participants identify and overcome emotional problems and demonstrate how patients can develop good coping skills for good mental health. It is a modular program developed by the Centre for Mental Health Research at the Australian National University [58]. Each module explores topics including the following: why someone feels the way they do, changing the way they think, changing “warped” thoughts, knowing what makes an individual upset, assertiveness, and interpersonal skills training [58]. Once registered, individuals work through a series of modules or workbooks, which can be undertaken piecemeal depending on the time available. Many studies have demonstrated the effectiveness of MoodGYM for MDD and anxiety in both outpatients and inpatients in different clinical settings [19,21,59-67]. In addition, it is effective for the mitigation of burnout, depression, and suicidality among health care students and professionals [68].

### Objectives

The goal of this project is to evaluate the initial comparative clinical effectiveness of rTMS treatments when used with and without iCBT.

Due to the limited availability of data in this specific area, another goal of the study is to generate effect size data for these interventions, which will help inform sample size and power calculations for a full randomized clinical trial. Patient outcomes are organized according to recovery variables (eg, recovery and stigma), functional variables (quality of life and employment), symptom variables (psychological symptoms and overall outcomes), and service variables (eg, health service utilization, cost, and satisfaction).

## Methods

### Ethics and Dissemination

The study will be conducted per the Declaration of Helsinki (Hong Kong Amendment) and the Canadian guidelines for Good Clinical Practice. All participants will provide informed consent before study inclusion. The results will be disseminated at several levels, including participants, practitioners, academics/researchers, and health care organizations.

The study will be a prospective, parallel design, two-arm, rater-blinded randomized controlled pilot trial with a recruitment period of 12 months. It will involve active treatment for six weeks and an observation period of 6 months for each participant. An overview of the timeline for the project is in Table 1. The research will be carried out in an Addiction and Mental Health clinic in a large, sociodemographically diverse city in Western Canada (Edmonton, Alberta).

**Table 1:** Gantt chart timeline.

Milestones		Year 1							Year 2			
		Q1	Q2		Q3		Q4		Q1		Q2	
**Milestone 1: Recruiting and training of trainee in psychiatry, setting up of infrastructure for iCBT^a^**												
	1.1. Advertising and recruitment of a trainee in psychiatry who will support the research/evaluation of the project component, apply rTMS^b,^ and facilitate iCBT.	✓										
**Milestone 2: The recruitment of study participants**												
	2.1. Recruitment, baseline assessment, and randomization			✓		✓						
	2.2. Assignment into one of the two arms of the study			✓		✓						
	2.3. Delivery of iCBT and rTMS to participants			✓		✓		✓				
**Milestone 3: Follow-up assessment of study participants**												
	3.1. Follow-up assessments of individual study participants							✓		✓		
	3.2. Follow-up satisfaction survey of participants, all groups							✓		✓		
**Milestone 4: Data compilation, data analysis, and preparation of reports, publications, and presentations**												
	4.1. Data compilation			✓		✓		✓		✓		✓
	4.2. Data analysis			✓		✓		✓		✓		✓
	4.3. Preparation of reports, publications, and presentations											✓

^a^iCBT: internet-based cognitive-behavioral therapy.

^b^rTMS: repetitive transcranial magnetic stimulation.

### Inclusion Criteria

Study participants should meet the following inclusion criteria:

- Aged 18-65 years
- Suffering from a major depressive episode based on Diagnostic and Statistical Manual of Mental Disorders (DSM) 5 criteria and having failed two or more standard antidepressant treatments during the current episode.
- Hamilton Depression Rating Scale (17-HAM-D) score of 10 or more
- Participant may be on psychotropic medications including antidepressants, antipsychotics, benzodiazepines, and anticonvulsants
- Have a good understanding of the English language with fair computer/internet skills, and able and willing to provide informed consent.

### Exclusion Criteria

The exclusion criteria for this study are the following:

- Diagnosis with the following conditions (current unless otherwise stated):
-
  - A neurological disorder, including a history of seizures, cerebrovascular disease, primary or secondary tumors in the central nervous system, stroke, cerebral aneurysm, movement disorder, or any lifetime history of loss of consciousness due to head injury.
  - Any current Axis 1 psychotic disorder (including substance-induced psychosis, psychotic disorder due to a medical condition, or major depression with psychotic features), as defined by the Mini-International Neuropsychiatric Interview [69] at the screening visit.
  - Any current Axis II personality disorder that would interfere with participation in the study or might affect cognition and ability to participate meaningfully, as well as mental retardation identified through medical history or by the investigator.
  - A current amnestic disorder, dementia, or delirium as defined by a Montreal Cognitive Assessment score of ≤16, or any other neurological or mental disease that might affect cognition or the ability to participate in CBT meaningfully.
- Participation in any drug or device clinical trial in the six weeks (42 days) prior to the screening visit and/or participation in another clinical trial for the duration of the study.
- Participants who are pregnant/breastfeeding.
- Discovery and/or the sudden appearance of any condition or circumstance from the above list that, in the opinion of the investigator, has the potential to prevent study completion and/or to have a confounding effect on outcome assessments.

The rTMS-trained health care practitioners’ team will determine a participant’s eligibility for the rTMS treatments. Once the individual has been accepted into the rTMS program, a member of our research team will introduce the study to him/her, give them a copy of the information leaflet, and ask if they would also be interested in enrolling in our study. The recruitment and an informed consent process will involve a face-to-face meeting with the eligible participant during the week of their rTMS eligibility assessment, which occurs 1 week before beginning the rTMS sessions. Participants can also withdraw from the study at any time without providing a reason. To withdraw, participants can contact the research coordinator to let him/her know. If participants leave the study, we will not collect new health information about them, and they may ask the research coordinator to withdraw any data we have already collected from them before data analysis and dissemination.

### Interventions

Participants would be randomly assigned to receive either rTMS alone or rTMS plus iCBT. Participants in both arms of the study will attend an introductory visit to introduce the rTMS system to them and explain the procedure that will be carried out in each visit. Participants will be asked to complete standard questionnaires as part of their participation in the rTMS program. A week before the start of rTMS sessions, the participants will be invited into the clinic for motor threshold (MT) assessments, which are important for selection of stimulation intensities for each patient, and assessment for inclusion in the study. MT is roughly a measure of the TMS intensity necessary to evoke a peripheral motor response. These assessments will be done by the rTMS team, which includes health care practitioners trained on how to assess and use rTMS. Each of the assessments will take 3-5 minutes, and the total time will be 35-45 minutes. The timeline for visits will be the same for all participants. All participants will be scheduled to receive 30 sessions of rTMS treatments over 6 weeks as predetermined by Alberta Health Services' Strategic Clinical Network for Addiction and Mental Health. In addition, participants in the rTMS plus iCBT arm of the study will be assisted in registering for the iCBT program (MoodGYM) to receive unique login information. They will be assisted in participating in 12 one-hour sessions of iCBT at the clinic followed by rTMS treatments on the same day. These in-clinic iCBT sessions will be scheduled at about three-day intervals (ideally Tuesdays and Thursdays) so that participants receive two iCBT sessions each week. These in-clinic iCBT sessions are necessary to avoid poor treatment adherence and high discontinuation rates, as conducting these sessions by themselves at home may represent a major challenge for patients with TRD. Participants would also be encouraged to continue with iCBT treatments on their own at home, outside the sessions delivered in the clinic. The personal information relating to the MoodGYM website that will be collected consists of age group, gender, email address, password, answers to secret questions nominated, and the information the participants submit when using the MoodGYM website (including quizzes, workbooks, and diaries). In addition, the following information about participants’ usage of the MoodGYM website will be collected by using transient cookies: participants’ browser’s internet address, the date and time the site was visited, the pages that were accessed and the documents that were downloaded, the type of browser used, the number of bookmarks created, the last viewed date, the time of visit, and details about participant’s subscription excluding credit card details. MoodGYM has its own privacy policy that controls the personal information obtained from all participants under their respective User Data profile. There is no risk that a participant’s diagnosis could be exposed to the public should a breach at MoodGYM occur.

All participants will be followed up for 6 months and will be encouraged to continue to receive whatever community clinic/program treatments or supports are part of their usual care.

### Sample Size

Consistent with the idea that this is a pilot study, with no established effect size data available to aid in power and sample size calculations, the research will use data that can be elicited from participants who can be enrolled within existing operational resources. This method is acceptable for pilot studies involving novel interventions and has been described by Haynes et al [70] as using “the participants I can get.” Therefore, the study will be limited to a sample size of 100, with about 50 participants recruited into each arm of the study. Patients with TRD are vulnerable to severe depressive attacks, and it can reasonably be expected that only a small number of eligible participants will enroll in and complete the study.

## Results

We hypothesize that participants enrolled in the rTMS plus iCBT treatment arm of the study will achieve superior outcomes compared with participants enrolled in the rTMS alone arm of the study on each outcome measure used.

### Outcomes

Outcome measures and time points are detailed in Table 2 and follow from the aim and objectives of the study. All measures (except patient experience questionnaire, interviews, and data extraction) are objective measures with published information regarding reliability and validity. The Hamilton Depression Rating Scale (HAM-D) [71,72] will be the primary outcome and all other measures will be secondary outcomes. These measures include the following: Columbia Suicide Severity Rating Scale (CSSRS) [73,74], Young Mania Rating Scale (YMRS) [75], Quick Inventory of Depressive Symptomatology Self Report-16 (QIDS SR-16) [76], Frequency, Intensity, and Burden of Side Effects Ratings (FIBSER; edited for rTMS) [77], Patient Rated Inventory of Side Effects (PRISE) [78], EuroQoL 5-Dimension 5-Level (EQ-5D-5L) [79,80], and World Health Organization Disability Assessment 2.0 (WHODAS 2.0) [81]. The primary outcome measure will be the mean change in the scores on the Hamilton Depression Rating Scale. Patient service utilization data and clinician-rated measures will also be used to gauge patient progress. Patient data will be analyzed with descriptive statistics, repeated measures, and correlational analyses. All quantitative data will be analyzed using SPSS (Version 26; IBM Corp) [82].

**Table 2:** Client-oriented outcome measures.

Outcome measures				Time points assessed			
Variable type and construct		Tool	Rater	Baseline	1 month	3 months	6 months
**Symptom variables**							
	Depression	Hamilton Depression Rating Scale (HAM-D)	Clinician	✓	✓	✓	✓
	Depression	Quick Inventory of Depressive Symptomatology Self Report-16 (QIDS SR-16)	Client	✓	✓	✓	✓
	Suicidal ideation	Columbia Suicide Severity Rating Scale (CSSRS)	Clinician	✓	✓	✓	✓
	Mania	Young Mania Rating Scale (YMRS)	Clinician	✓	✓	✓	✓
**Functional variables**							
	Side effects	Frequency, Intensity, and Burden of Side Effects Ratings (FIBSER; edited for rTMS)	Client	✓	✓	✓	✓
	Side effects	Patient Rated Inventory of Side Effects (PRISE)	Client	✓	✓	✓	✓
	Disability measures	World Health Organization Disability Assessment 2.0 (WHODAS 2.0)	Client	✓	✓	✓	✓
	Quality of life	EuroQoL 5-Dimension 5-Level (EQ-5D-5L)	Client	✓	✓	✓	✓

### Randomization and Blinding

A simple randomization technique will be used based on a single sequence of random assignments. A computer-generated Excel sheet (Microsoft Corp) will be used for simple randomization of subjects. Randomization will be stratified by using permuted blocks to ensure balance (1:1) between the two follow-up treatment groups. The randomization codes will be transmitted by an independent statistician via text message directly to a researcher’s password-protected phone line with a secure online backup. This will commence as soon as participants sign the consent forms.

As it will not be possible for participants to be blinded, treatment allocation will be made explicit to them as soon as randomization is concluded. Primary outcome assessors will be blinded to treatment group allocation by not involving them in discussions about study participants and not granting them access to the database that contains the randomization code. After data collection is complete, all data will undergo a blind review for the purposes of finalizing the planned analysis.

### Follow-up Assessment

At 1, 3, and 6 months, a blinded researcher will contact all study participants and help them complete a range of assessment tools relating to the primary and secondary outcome measures. They will be offered the opportunity to complete the assessments face-to-face or over the phone. Qualitative data collection will be in the form of a patient experience questionnaire and a focus group workshop, which will be conducted at 3 and 6 months. At 6 months, data related to each person’s clinic/program attendance rates and utilization of health services will be compiled from administrative records by the blinded researcher.

### Patient and Public Involvement

This study was designed to address the clinical urgency to identify and respond to early evidence of treatment resistance using treatments that have proven efficacy in these more difficult-to-treat psychiatric patients. The study is designed as patient-oriented research with the active involvement of a patient representative who will be a coauthor of the study protocol. Our randomized trial offers participants the opportunity to provide feedback regarding the burden of the intervention through a focused group workshop involving a cross-section of participants from the two arms of the study.

### Ethics and Dissemination

The study will be conducted per the Declaration of Helsinki (Hong Kong Amendment) and Good Clinical Practice (Canadian Guidelines). Written informed consent will be obtained from each participant. The study has received ethical clearance from the Health Ethics Research Board of the University of Alberta (Pro00094208). The study is registered with ClinicalTrials.gov (registration number NCT04239651; preresult). The study results, expected 18 months after commencement of recruitment, will be disseminated at several levels, including participants, practitioners, academics/researchers, and health care organizations.

The investigator’s team will plan an organizational engagement strategy to advance discussions about practicability and effectiveness before the conclusion of the trial. This will help ensure the findings are a relevant part of decision-making processes in a way that is aligned with study findings as they emerge. This may facilitate the planning of a more extensive study that is endorsed at both leadership and operational levels so that the potential benefits of the interventions can reach participants in a timelier fashion.

## Discussion

### Overview

The results of the study will provide the data required to evaluate the initial effectiveness of rTMS plus iCBT for patients diagnosed with resistant depression. The majority of RCTs support the efficacy of rTMS for major depression. The data collected on rTMS is significant only as a single intervention. The concomitant application of psychotherapy with rTMS has not been investigated previously. We hope that this project will provide us with a concrete base of data to evaluate the practical application and efficacy of using a novel combination of these two treatment modalities (rTMS plus iCBT). To our knowledge, no clinical trials have applied these two new treatment interventions together before. Due to the limited availability of data in this specific area, another aim is to generate effect size data for these interventions, which will help in sample size and power calculations for a full randomized clinical trial.

### Strengths of This Study

The strengths of this study include the following:

- Randomization of participants will ensure that participants in the two treatment arms have somewhat similar psychiatric morbidity at baseline.
- Blinding of primary outcome assessors for the primary outcome measures will ensure the elimination of bias in outcome measures.

### Limitation of This Study

The limitations of this study include the following:

- The small sample size may reduce the study power, which will limit the ability of the study to detect differences in outcome measures between participants in the two treatment arms.
- Possible variability in concomitant treatments (medication and/or psychotherapy) being received by patients outside the rTMS clinic as well as the differing lengths of treatment time between the two arms of the study could have confounding effects on the outcomes of our interventions.

## Abbreviations

CAM: complementary and alternative medicine
CANMAT: Canadian Network for Mood and Anxiety Treatments
CBT: cognitive-behavioral therapy
DLPFC: left dorsolateral prefrontal cortex
DSM: Diagnostic and Statistical Manual of Mental Disorders
ECT: electroconvulsive therapy
iCBT: internet-delivered cognitive-behavioral therapy
MDD: major depressive disorder
rTMS: repetitive transcranial magnetic stimulation
TRD: treatment-resistant depression

## References

[1] Kessler RC, Berglund P, Demler O, Jin R, Koretz D, Merikangas KR, Rush AJ, Walters EE, Wang PS, National CSR (2003). The epidemiology of major depressive disorder: results from the National Comorbidity Survey Replication (NCS-R). JAMA.

[2] Murray CA, Lopez AD (1996). The global burden of disease: a comprehensive assessment of mortality and disability from diseases, injuries, and risk factors in 1990 and projected to 2020.

[3] American Foundation for Suicide Prevention (2009). You are not alone. Mental health conditions and suicide.

[4] Sackeim HA (2001). The definition and meaning of treatment-resistant depression. J Clin Psychiatry.

[5] Rush AJ, Thase ME, Dubé S (2003). Research issues in the study of difficult-to-treat depression. Biol Psychiatry.

[6] Greden JF (2002). Unmet need: what justifies the search for a new antidepressant?. J Clin Psychiatry.

[7] Papakostas GI (2008). Tolerability of modern antidepressants. J Clin Psychiatry.

[8] Leon AC, Solomon DA, Mueller TI, Endicott J, Rice JP, Maser JD, Coryell W, Keller MB (2003). A 20-year longitudinal observational study of somatic antidepressant treatment effectiveness. Am J Psychiatry.

[9] Prudic J, Haskett RF, Mulsant B, Malone KM, Pettinati HM, Stephens S, Greenberg R, Rifas SL, Sackeim HA (1996). Resistance to antidepressant medications and short-term clinical response to ECT. Am J Psychiatry.

[10] Nierenberg AA, Fava M, Trivedi MH, Wisniewski SR, Thase ME, McGrath PJ, Alpert JE, Warden D, Luther JF, Niederehe G, Lebowitz B, Shores-Wilson K, Rush AJ (2006). A comparison of lithium and T(3) augmentation following two failed medication treatments for depression: a STAR*D report. Am J Psychiatry.

[11] Fava M, Rush AJ, Wisniewski SR, Nierenberg AA, Alpert JE, McGrath PJ, Thase ME, Warden D, Biggs M, Luther JF, Niederehe G, Ritz L, Trivedi MH (2006). A comparison of mirtazapine and nortriptyline following two consecutive failed medication treatments for depressed outpatients: a STAR*D report. Am J Psychiatry.

[12] McGrath PJ, Stewart JW, Fava M, Trivedi MH, Wisniewski SR, Nierenberg AA, Thase ME, Davis L, Biggs MM, Shores-Wilson K, Luther JF, Niederehe G, Warden D, Rush AJ (2006). Tranylcypromine versus venlafaxine plus mirtazapine following three failed antidepressant medication trials for depression: a STAR*D report. Am J Psychiatry.

[13] Trivedi MH, Rush AJ, Wisniewski SR, Nierenberg AA, Warden D, Ritz L, Norquist G, Howland RH, Lebowitz B, McGrath PJ, Shores-Wilson K, Biggs MM, Balasubramani GK, Fava M, STAR* DT (2006). Evaluation of outcomes with citalopram for depression using measurement-based care in STAR*D: implications for clinical practice. Am J Psychiatry.

[14] Rush AJ, Trivedi MH, Wisniewski SR, Stewart JW, Nierenberg AA, Thase ME, Ritz L, Biggs MM, Warden D, Luther JF, Shores-Wilson K, Niederehe G, Fava M, STAR*D Study Team (2006). Bupropion-SR, sertraline, or venlafaxine-XR after failure of SSRIs for depression. N Engl J Med.

[15] Trivedi MH, Fava M, Wisniewski SR, Thase ME, Quitkin F, Warden D, Ritz L, Nierenberg AA, Lebowitz BD, Biggs MM, Luther JF, Shores-Wilson K, Rush AJ, STAR*D Study Team (2006). Medication augmentation after the failure of SSRIs for depression. N Engl J Med.

[16] Rush AJ, Trivedi MH, Wisniewski SR, Nierenberg AA, Stewart JW, Warden D, Niederehe G, Thase ME, Lavori PW, Lebowitz BD, McGrath PJ, Rosenbaum JF, Sackeim HA, Kupfer DJ, Luther J, Fava M (2006). Acute and longer-term outcomes in depressed outpatients requiring one or several treatment steps: a STAR*D report. Am J Psychiatry.

[17] Lam RW, McIntosh D, Wang J, Enns MW, Kolivakis T, Michalak EE, Sareen J, Song W, Kennedy SH, MacQueen GM, Milev RV, Parikh SV, Ravindran AV, CANMAT Depression Work Group (2016). Canadian Network for Mood and Anxiety Treatments (CANMAT) 2016 Clinical Guidelines for the Management of Adults with Major Depressive Disorder: Section 1. Disease Burden and Principles of Care. Can J Psychiatry.

[18] Lal S, Adair CE (2014). E-mental health: a rapid review of the literature. Psychiatr Serv.

[19] Karasouli E, Adams A (2014). Assessing the Evidence for e-Resources for Mental Health Self-Management: A Systematic Literature Review. JMIR Ment Health.

[20] Grist R, Porter J, Stallard P (2017). Mental Health Mobile Apps for Preadolescents and Adolescents: A Systematic Review. J Med Internet Res.

[21] Andrews G, Basu A, Cuijpers P, Craske MG, McEvoy P, English CL, Newby JM (2018). Computer therapy for the anxiety and depression disorders is effective, acceptable and practical health care: An updated meta-analysis. J Anxiety Disord.

[22] Arnberg FK, Linton SJ, Hultcrantz M, Heintz E, Jonsson U (2014). Internet-delivered psychological treatments for mood and anxiety disorders: a systematic review of their efficacy, safety, and cost-effectiveness. PLoS One.

[23] Vallury KD, Jones M, Oosterbroek C (2015). Computerized Cognitive Behavior Therapy for Anxiety and Depression in Rural Areas: A Systematic Review. J Med Internet Res.

[24] Titov N (2011). Internet-delivered psychotherapy for depression in adults. Curr Opin Psychiatry.

[25] Richards D, Richardson T (2012). Computer-based psychological treatments for depression: a systematic review and meta-analysis. Clin Psychol Rev.

[26] Eells TD, Barrett MS, Wright JH, Thase M (2014). Computer-assisted cognitive-behavior therapy for depression. Psychotherapy (Chic).

[27] Renton T, Tang H, Ennis N, Cusimano MD, Bhalerao S, Schweizer TA, Topolovec-Vranic J (2014). Web-based intervention programs for depression: a scoping review and evaluation. J Med Internet Res.

[28] Holländare F, Anthony SA, Randestad M, Tillfors M, Carlbring P, Andersson G, Engström I (2013). Two-year outcome of internet-based relapse prevention for partially remitted depression. Behav Res Ther.

[29] Patten SB, Kennedy SH, Lam RW, O'Donovan C, Filteau MJ, Parikh SV, Ravindran AV (2009). Canadian Network for Mood and Anxiety Treatments (CANMAT) Clinical Guidelines for the Management of Major Depressive Disorder in Adults. I. Classification, Burden and Principles of Management. Journal of Affective Disorders.

[30] Patten SB (2016). Updated CANMAT Guidelines for Treatment of Major Depressive Disorder. Can J Psychiatry.

[31] Parikh SV, Quilty LC, Ravitz P, Rosenbluth M, Pavlova B, Grigoriadis S, Velyvis V, Kennedy SH, Lam RW, MacQueen GM, Milev RV, Ravindran AV, Uher R, CANMAT Depression Work Group (2016). Canadian Network for Mood and Anxiety Treatments (CANMAT) 2016 Clinical Guidelines for the Management of Adults with Major Depressive Disorder: Section 2. Psychological Treatments. Can J Psychiatry.

[32] Kennedy SH, Lam RW, McIntyre RS, Tourjman SV, Bhat V, Blier P, Hasnain M, Jollant F, Levitt AJ, MacQueen GM, McInerney SJ, McIntosh D, Milev RV, Müller DJ, Parikh SV, Pearson NL, Ravindran AV, Uher R, CANMAT Depression Work Group (2016). Canadian Network for Mood and Anxiety Treatments (CANMAT) 2016 Clinical Guidelines for the Management of Adults with Major Depressive Disorder: Section 3. Pharmacological Treatments. Can J Psychiatry.

[33] Milev RV, Giacobbe P, Kennedy SH, Blumberger DM, Daskalakis ZJ, Downar J, Modirrousta M, Patry S, Vila-Rodriguez F, Lam RW, MacQueen GM, Parikh SV, Ravindran AV, CANMAT Depression Work Group (2016). Canadian Network for Mood and Anxiety Treatments (CANMAT) 2016 Clinical Guidelines for the Management of Adults with Major Depressive Disorder: Section 4. Neurostimulation Treatments. Can J Psychiatry.

[34] Ravindran AV, Balneaves LG, Faulkner G, Ortiz A, McIntosh D, Morehouse RL, Ravindran L, Yatham LN, Kennedy SH, Lam RW, MacQueen GM, Milev RV, Parikh SV, CANMAT Depression Work Group (2016). Canadian Network for Mood and Anxiety Treatments (CANMAT) 2016 Clinical Guidelines for the Management of Adults with Major Depressive Disorder: Section 5. Complementary and Alternative Medicine Treatments. Can J Psychiatry.

[35] MacQueen GM, Frey BN, Ismail Z, Jaworska N, Steiner M, Lieshout RJV, Kennedy SH, Lam RW, Milev RV, Parikh SV, Ravindran AV, CANMAT Depression Work Group (2016). Canadian Network for Mood and Anxiety Treatments (CANMAT) 2016 Clinical Guidelines for the Management of Adults with Major Depressive Disorder: Section 6. Special Populations: Youth, Women, and the Elderly. Can J Psychiatry.

[36] Hallett M (2007). Transcranial magnetic stimulation: a primer. Neuron.

[37] Noda Y, Silverstein WK, Barr MS, Vila-Rodriguez F, Downar J, Rajji TK, Fitzgerald PB, Mulsant BH, Vigod SN, Daskalakis ZJ, Blumberger DM (2015). Neurobiological mechanisms of repetitive transcranial magnetic stimulation of the dorsolateral prefrontal cortex in depression: a systematic review. Psychol Med.

[38] Galletly C, Gill S, Clarke P, Burton C, Fitzgerald PB (2012). A randomized trial comparing repetitive transcranial magnetic stimulation given 3 days/week and 5 days/week for the treatment of major depression: is efficacy related to the duration of treatment or the number of treatments?. Psychol Med.

[39] McGirr A, Van den Eynde F, Tovar-Perdomo S, Fleck MPA, Berlim MT (2015). Effectiveness and acceptability of accelerated repetitive transcranial magnetic stimulation (rTMS) for treatment-resistant major depressive disorder: an open label trial. J Affect Disord.

[40] George MS, Raman R, Benedek DM, Pelic CG, Grammer GG, Stokes KT, Schmidt M, Spiegel C, Dealmeida N, Beaver KL, Borckardt JJ, Sun X, Jain S, Stein MB (2014). A two-site pilot randomized 3 day trial of high dose left prefrontal repetitive transcranial magnetic stimulation (rTMS) for suicidal inpatients. Brain Stimul.

[41] McDonald WM, Durkalski V, Ball ER, Holtzheimer PE, Pavlicova M, Lisanby SH, Avery D, Anderson BS, Nahas Z, Zarkowski P, Sackeim HA, George MS (2011). Improving the antidepressant efficacy of transcranial magnetic stimulation: maximizing the number of stimulations and treatment location in treatment-resistant depression. Depress Anxiety.

[42] Carpenter LL, Janicak PG, Aaronson ST, Boyadjis T, Brock DG, Cook IA, Dunner DL, Lanocha K, Solvason HB, Demitrack MA (2012). Transcranial magnetic stimulation (TMS) for major depression: a multisite, naturalistic, observational study of acute treatment outcomes in clinical practice. Depress Anxiety.

[43] Kedzior KK, Azorina V, Reitz SK (2014). More female patients and fewer stimuli per session are associated with the short-term antidepressant properties of repetitive transcranial magnetic stimulation (rTMS): a meta-analysis of 54 sham-controlled studies published between 1997-2013. Neuropsychiatr Dis Treat.

[44] Berlim MT, van den Eynde F, Tovar-Perdomo S, Daskalakis ZJ (2014). Response, remission and drop-out rates following high-frequency repetitive transcranial magnetic stimulation (rTMS) for treating major depression: a systematic review and meta-analysis of randomized, double-blind and sham-controlled trials. Psychol Med.

[45] Hovington CL, McGirr A, Lepage M, Berlim MT (2013). Repetitive transcranial magnetic stimulation (rTMS) for treating major depression and schizophrenia: a systematic review of recent meta-analyses. Ann Med.

[46] Leggett LE, Soril LJJ, Coward S, Lorenzetti DL, MacKean G, Clement FM (2015). Repetitive Transcranial Magnetic Stimulation for Treatment-Resistant Depression in Adult and Youth Populations: A Systematic Literature Review and Meta-Analysis. Prim Care Companion CNS Disord.

[47] Gaynes BN, Lloyd SW, Lux L, Gartlehner G, Hansen RA, Brode S, Jonas DE, Swinson Evans T, Viswanathan M, Lohr KN (2014). Repetitive transcranial magnetic stimulation for treatment-resistant depression: a systematic review and meta-analysis. J Clin Psychiatry.

[48] Health Quality Ontario (2016). Repetitive Transcranial Magnetic Stimulation for Treatment-Resistant Depression: A Systematic Review and Meta-Analysis of Randomized Controlled Trials. Ont Health Technol Assess Ser.

[49] Berlim MT, Van den Eynde F, Jeff Daskalakis Z (2013). Clinically meaningful efficacy and acceptability of low-frequency repetitive transcranial magnetic stimulation (rTMS) for treating primary major depression: a meta-analysis of randomized, double-blind and sham-controlled trials. Neuropsychopharmacology.

[50] Cohen RB, Boggio PS, Fregni F (2009). Risk factors for relapse after remission with repetitive transcranial magnetic stimulation for the treatment of depression. Depress Anxiety.

[51] Dunner DL, Aaronson ST, Sackeim HA, Janicak PG, Carpenter LL, Boyadjis T, Brock DG, Bonneh-Barkay D, Cook IA, Lanocha K, Solvason HB, Demitrack MA (2014). A multisite, naturalistic, observational study of transcranial magnetic stimulation for patients with pharmacoresistant major depressive disorder: durability of benefit over a 1-year follow-up period. J Clin Psychiatry.

[52] Janicak PG, Nahas Z, Lisanby SH, Solvason HB, Sampson SM, McDonald WM, Marangell LB, Rosenquist P, McCall WV, Kimball J, O'Reardon JP, Loo C, Husain MH, Krystal A, Gilmer W, Dowd SM, Demitrack MA, Schatzberg AF (2010). Durability of clinical benefit with transcranial magnetic stimulation (TMS) in the treatment of pharmacoresistant major depression: assessment of relapse during a 6-month, multisite, open-label study. Brain Stimul.

[53] Richieri R, Guedj E, Michel P, Loundou A, Auquier P, Lançon C, Boyer L (2013). Maintenance transcranial magnetic stimulation reduces depression relapse: a propensity-adjusted analysis. J Affect Disord.

[54] Fitzgerald PB, Grace N, Hoy KE, Bailey M, Daskalakis ZJ (2013). An open label trial of clustered maintenance rTMS for patients with refractory depression. Brain Stimul.

[55] Hazlett‐Stevens H, Craske MG, Bond FW, Craske MG (2002). Brief cognitive-behavioral therapy: definition and scientific foundations. Handbook of Brief Cognitive Behaviour Therapy.

[56] Gratzer D, Khalid-Khan F (2016). Internet-delivered cognitive behavioural therapy in the treatment of psychiatric illness. CMAJ.

[57] Olthuis JV, Watt MC, Bailey K, Hayden JA, Stewart SH (2015). Therapist-supported Internet cognitive behavioural therapy for anxiety disorders in adults. Cochrane Database Syst Rev.

[58] Haldane D (2006). MoodGYM.

[59] Kaltenthaler E, Shackley P, Stevens K, Beverley C, Parry G, Chilcott J (2002). A systematic review and economic evaluation of computerised cognitive behaviour therapy for depression and anxiety. Health Technol Assess.

[60] Kaltenthaler E, Brazier J, De NE, Tumur I, Ferriter M, Beverley C, Parry G, Rooney G, Sutcliffe P (2006). Computerised cognitive behaviour therapy for depression and anxiety update: a systematic review and economic evaluation. Health Technol Assess.

[61] Kaltenthaler E, Parry G, Beverley C (2004). Computerized Cognitive Behaviour Therapy: A Systematic Review. Behav Cogn Psychother.

[62] Kaltenthaler E, Parry G, Beverley C, Ferriter M (2008). Computerised cognitive-behavioural therapy for depression: systematic review. Br J Psychiatry.

[63] Spek V, Cuijpers P, Nyklícek I, Riper H, Keyzer J, Pop V (2007). Internet-based cognitive behaviour therapy for symptoms of depression and anxiety: a meta-analysis. Psychol Med.

[64] Andersson G, Cuijpers P (2009). Internet-based and other computerized psychological treatments for adult depression: a meta-analysis. Cogn Behav Ther.

[65] Griffiths K, Christensen H (2006). Review of randomised controlled trials of Internet interventions for mental disorders and related conditions. Clinical Psychologist.

[66] Griffiths KM, Farrer L, Christensen H (2010). The efficacy of internet interventions for depression and anxiety disorders: a review of randomised controlled trials. Med J Aust.

[67] Wade AG (2010). Use of the internet to assist in the treatment of depression and anxiety: a systematic review. Prim Care Companion J Clin Psychiatry.

[68] Pospos S, Young IT, Downs N, Iglewicz A, Depp C, Chen JY, Newton I, Lee K, Light GA, Zisook S (2017). Web-Based Tools and Mobile Applications To Mitigate Burnout, Depression, and Suicidality Among Healthcare Students and Professionals: a Systematic Review. Acad Psychiatry.

[69] Sheehan DV, Lecrubier Y, Sheehan KH, Amorim P, Janavs J, Weiller E, Hergueta T, Baker R, Dunbar GC (1998). The Mini-International Neuropsychiatric Interview (M.I.N.I.): the development and validation of a structured diagnostic psychiatric interview for DSM-IV and ICD-10. J Clin Psychiatry.

[70] Haynes R, Guyatt G, Tugwell P (2005). Clinical epidemiology: how to do clinical practice research.

[71] Hamilton M (1960). A rating scale for depression. J Neurol Neurosurg Psychiatry.

[72] Carroll BJ, Fielding JM, Blashki TG (1973). Depression rating scales. A critical review. Arch Gen Psychiatry.

[73] Posner K, Brent D, Lucas C, Gould M, Stanley B, Brown G, Fisher P, Zelazny J, Burke A, Oquendo M, Mann J (2008). Columbia-suicide severity rating scale (C-SSRS).

[74] Shahid A, Wilkinson K, Marcu S, Shapiro C (2011). Columbia-Suicide Severity Rating Scale (C-SSRS). STOP, THAT and One Hundred Other Sleep Scales.

[75] Young R, Biggs J, Ziegler V, Meyer D (1978). A rating scale for mania: reliability, validity and sensitivity. Br J Psychiatry.

[76] Rush AJ, Trivedi MH, Ibrahim HM, Carmody TJ, Arnow B, Klein DN, Markowitz JC, Ninan PT, Kornstein S, Manber R, Thase ME, Kocsis JH, Keller MB (2003). The 16-Item Quick Inventory of Depressive Symptomatology (QIDS), clinician rating (QIDS-C), and self-report (QIDS-SR): a psychometric evaluation in patients with chronic major depression. Biol Psychiatry.

[77] Wisniewski SR, Rush AJ, Balasubramani GK, Trivedi MH, Nierenberg AA, STARD Investigators (2006). Self-rated global measure of the frequency, intensity, and burden of side effects. J Psychiatr Pract.

[78] Rush A, Asberg M (1999). Unpublished rating scale. Patient Rated Inventory of Side Effects (PRISE).

[79] Szende A, Williams A (2004). Measuring self-reported population health: An international perspective based on EQ-5D.

[80] Brooks R, Rabin R, De Charro F (2013). The measurement and valuation of health status using EQ-5D: a European perspective. Evidence from the EuroQol BIOMED Research Programme.

[81] Ustün TB, Chatterji S, Kostanjsek N, Rehm J, Kennedy C, Epping-Jordan J, Saxena S, von Korff M, Pull C, WHO/NIH Joint Project (2010). Developing the World Health Organization Disability Assessment Schedule 2.0. Bull World Health Organ.

[82] IBM Corp IBM SPSS Statistics for Macintosh, Version 26.0.

